# A Focus on the Cerebellum: From Embryogenesis to an Age-Related Clinical Perspective

**DOI:** 10.3389/fnsys.2021.646052

**Published:** 2021-04-09

**Authors:** Greta Amore, Giulia Spoto, Antonio Ieni, Luigi Vetri, Giuseppe Quatrosi, Gabriella Di Rosa, Antonio Gennaro Nicotera

**Affiliations:** ^1^Unit of Child Neurology and Psychiatry, Department of Human Pathology of the Adult and Developmental Age “Gaetano Barresi”, University of Messina, Messina, Italy; ^2^Unit of Pathology, Department of Human Pathology of the Adult and Developmental Age “Gaetano Barresi”, University of Messina, Messina, Italy; ^3^Department of Health Promotion, Mother and Child Care, Internal Medicine and Medical Specialties, University of Palermo, Palermo, Italy

**Keywords:** age-related clinical findings, anatomy, cerebellar, cerebellum, circuitry, neurodevelopment, neuroimaging, neurophysiology

## Abstract

The cerebellum and its functional multiplicity and heterogeneity have been objects of curiosity and interest since ancient times, giving rise to the urge to reveal its complexity. Since the first hypothesis of cerebellar mere role in motor tuning and coordination, much more has been continuously discovered about the cerebellum’s circuitry and functioning throughout centuries, leading to the currently accepted knowledge of its prominent involvement in cognitive, social, and behavioral areas. Particularly in childhood, the cerebellum may subserve several age-dependent functions, which might be compromised in several Central Nervous System pathologies. Overall, cerebellar damage may produce numerous signs and symptoms and determine a wide variety of neuropsychiatric impairments already during the evolutive age. Therefore, an early assessment in children would be desirable to address a prompt diagnosis and a proper intervention since the first months of life. Here we provide an overview of the cerebellum, retracing its morphology, histogenesis, and physiological functions, and finally outlining its involvement in typical and atypical development and the age-dependent patterns of cerebellar dysfunctions.

## Introduction: The Cerebellum Through History

The cerebellum, also known as “little brain,” has been an object of interest and research for centuries. Throughout history, many prominent personalities, as recently discussed by Voogd and Koehler (2018), have been trying to reveal the cerebellum complexity to achieve a better understanding of this peculiar structure. Among those who contributed in implementing the knowledge on structural and functional aspects of the cerebellum, it is worth mentioning the French anatomist Vieussens (1641–1715), whose compendium “Neurographia Universalis” (Vieussens, 1685), written in Latin, provided an ahead of time description of the Central Nervous System (CNS; Manto and Huisman, 2018). This work, albeit still far from thorough, pointed out some relevant notions, such as the functional independence of the spinal cord from the rest of the CNS, and contributed in characterizing the *centrum ovale* (namely the central white matter of the cerebrum) and other structures, such as the “superior medullary velum” of the cerebellum, also known as “Vieussen’s valve” (JAMA, 1968; Loukas et al., 2007).

A major contribution to the topic came during the 18th century thanks to the work of the Italian professor of medicine, surgery, and obstetrics Vincenzo Malacarne, who, in his publications, among which is worth recalling “*Nuova esposizione della vera struttura del cervelletto umano”* (1776), provided a complete description of the human cerebellum anatomy (comprising the number of lobes and folia), introduced the terms tonsil, pyramid, lingual, and uvula, to date still in use and proposed a correlation between the number of cerebellar lamellae and the expression of intellectual faculties, hence asserting the existence of a strict relation between cerebellar underdevelopment and cretinism (Zanatta et al., 2018).

Further knowledge spread during the 19th century, through the meticulous research carried out by numerous scientists who contributed to improving the description of symptoms and signs of cerebellar lesions; for example, Rolando and Magendie emphasized the role of cerebellum in controlling, respectively, posture/movement and equilibrium, Luciani reported atonia, asthenia, astasia, and dysmetria as possible neurological cerebellar signs, Babinski described adiadochokinesia and pointed out the role of the cerebellum in synergia, while Sherrington linked the cerebellum to proprioception and modulation of reflexes (Manto and Huisman, 2018). Between 1917 and 1939, Holmes provided a thorough description of neurological signs and symptoms deriving from cerebellar lesions, such as disturbances of muscle tone (hypotonia) and voluntary movement, static tremor, asthenia and fatigability, astasia, vertigo, disturbances of ocular movements and nystagmus, abnormal speech, and reflexes (Holmes, 1917, 1939).

Innovations on the morphology and functionality of the cerebellum were continuously achieved throughout the 20th century, leading progressively to the currently accepted notions such as the division in 10 lobules, first proposed by Larsell (1970), the functional subdivision into a medial, an intermediate, and a lateral zone (Dow and Moruzzi, 1958), and cerebellar prominent involvement, not only in sensorimotor functions, but also in cognitive, social and behavioral areas (Roostaei et al., 2014).

## Anatomy of the Cerebellum

The cerebellum is a small structure of the hindbrain, weighing approximately from 136 to 169 *g* and representing about the 11% of brain weight in adult humans and 5–6% in neonates (Solov’ev, 2016). Despite its small size, it contains almost 80% of the global brain neurons and plays an important role in sensorimotor, cognitive, and affective functions (Roostaei et al., 2014).

### Cerebellar Gross Anatomy

The cerebellum is located in the posterior cranial fossa. It is separated, anteriorly, from the pons and the medulla oblongata by the fourth ventricle, and, superiorly, from the cerebrum by the Tentorium Cerebelli (an invagination of the dura mater). It globally presents two faces: the superior one is convex, crossed by the superior vermis and shows, laterally, the upper surfaces of the two cerebellar hemispheres; the inferior one is allocated in the posterior cranial fossa and presents a depression, in whose depth the inferior vermis is placed. A roughly ellipsoidal circumference separates these two faces, and opens anteriorly in the hilum of the cerebellum, from which the three cerebellar peduncles (superior, middle, and inferior) emerge. These latter represent the structures through which the afferents and efferences of the cerebellum pass and reach their targets (Voogd, 2003; Cattaneo, 2013; Roostaei et al., 2014).

**FIGURE 1:**
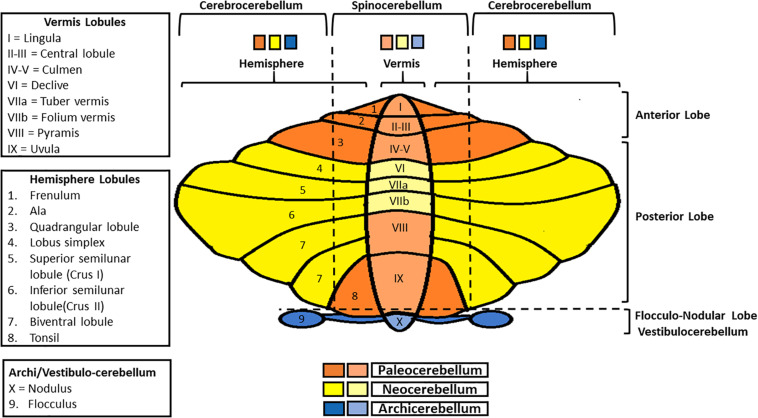
Schematic representation of cerebellar gross anatomy. Anterior-posteriorly, the cerebellum presents three lobes (anterior, posterior, and flocculo-nodular), each one subdivided in lobules. Medio-laterally, it is composed of a central part (the vermis), and two lateral ones (cerebellar hemispheres). Vermis and paravermial zones form the spinocerebellum, so-called since it communicates with the spinal cord; the most lateral zones of the hemispheres constitute the cerebrocerebellum, in connection with the cerebral cortex. Finally, in regard to phylogenesis, the cerebellum can be divided into three parts: the archicerebellum (the most ancient one, corresponding to the vestibulocerebellum), paleocerebellum and neocerebellum.

The cerebellum surface is globally composed of numerous parallel leaflike subdivisions, called folia (Voogd and Glickstein, 1998), giving it an onion-like aspect. Two main transversal fissures (the fissura prima, anteriorly, and the horizontal fissure, posteriorly) delineate three main lobes (the anterior lobe, the posterior lobe, and the flocculonodular lobe), each one subdivided in lobules (Figure 1; Larsell, 1970; Manni and Petrosini, 2004). Besides, considering the medio-lateral perspective, the cerebellum presents a central part, the vermis, and two lateral cerebellar hemispheres. Both the anterior and posterior lobes contain a part of the vermis and of the two hemispheres. Moreover, the flocculonodular lobe constitutes *per se* the so-called vestibulocerebellum/archicerebellum, the oldest one phylogenetically-wise (Manni and Petrosini, 2004). The medial zone (vermis) and the intermediate ones (paravermis) form the spinocerebellum, so called because of the sensorimotor afferents coming from the spinal cord. The lateral zones constitute the cerebrocerebellum, whose name is due to the presence of afferents/efferences from/to the cortex (Kandel et al., 2013; Roostaei et al., 2014).

Overall, on a phylogenetical basis, it is possible to distinguish among three parts: the archicerebellum, the paleocerebellum and the neocerebellum (Figure 1; Manni and Petrosini, 2004).

The archicerebellum is strictly connected with the vestibular nuclei (for this reason it also called “vestibulocerebellum”; Voogd et al., 1996), sending outputs to these structures directly, thus bypassing the deep nuclei (Roostaei et al., 2014). It is also involved in equilibrium, ocular movements, and vestibulo-ocular reflex regulation. The medial zone (nodulus) primarily controls the axial musculature, while the lateral parts (floccules) are mostly involved in eye pursuit movements and hand-eye coordination (Manni and Petrosini, 2004). Both the paleocerebellum and the neocerebellum include a part of the spinocerebellum and the cerebrocerebellum. Specifically, the paleocerebellum (corresponding to the anterior lobe), mostly regulates tone and posture, whereas the neocerebellum (corresponding to the posterior lobe) controls voluntary movements, as well as automatic and semi-automatic ones (Manni and Petrosini, 2004; Cattaneo, 2013; Roostaei et al., 2014).

The vermis receives visual, auditory and vestibular inputs, as well as sensorimotor ones from head, trunk and proximal portions of limbs, and it sends outputs, through the fastigial nucleus, to the cortex and the brainstem, generating the medial descending tracts (which control limbs and proximal muscles). The paramedian zones receive sensorimotor inputs from the distal parts of limbs and send outputs, through the interposed nucleus, to the corticospinal and rubrospinal systems, being in charge of the distal muscles of limbs and fingers. Overall, the outputs of these pathways are involved in postural control, balance, locomotion, and gaze direction (Manni and Petrosini, 2004; Kandel et al., 2013). Furthermore, they are also involved in adjusting motor outputs, integrating and comparing motor commands and sensorimotor feedback, and in the anticipatory control of posture and movements. The lateral zones receive from the cortex and send information, through the dentate nucleus, to motor, premotor and prefrontal cortices, being involved in motor planning and various cognitive tasks (Kandel et al., 2013). Nowadays, it is common knowledge that cerebellar dysfunctions may lead to the so-called “cerebellar cognitive affective syndrome” (CCAS), comprehensive of a wide variety of neurologic and psychiatric signs and symptoms, such as impaired executive functions, abnormal visuospatial cognition, language deficits, personality, and behavioral disorders (Schmahmann and Sherman, 1998).

### Cerebellar Microanatomy and Circuitry

The cerebellum presents an outer gray matter layer (namely the cerebellar cortex), a deeper cerebellar white matter (the so-called arbor vitae), and, within this latter, the deep cerebellar nuclei (dentate, globose, emboliform, and fastigial nuclei; Voogd, 2003).

**FIGURE 2:**
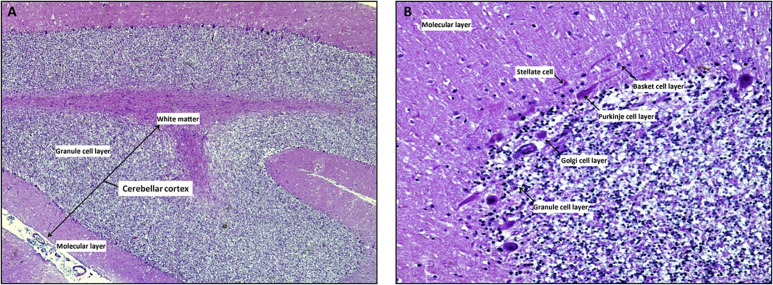
The cerebellar cortex. Mid-sagittal section reveals the three layers in cerebellar folia: the superficial molecular layer, the deepest called granular and the Purkinje cells layer at the interface between the granular and molecular layers Note the inner white core of white matter (**A**, x4 hematoxylin/eosin stain). At higher magnification, the molecular layer contains superficially located stellate cells, basket cells which are scattered among dendritic ramifications and numerous thin axons that run parallel to the long axis of the folia. Ganglionic or Purkinje cell layer is formed of a single row of Purkinje cells with large pear-shaped bodies; while the granular layer is composed by small granule cells with dark-staining nuclei/scanty cytoplasm and Golgi type II cells (**B**, x20 hematoxylin/eosin stain).

**FIGURE 3:**
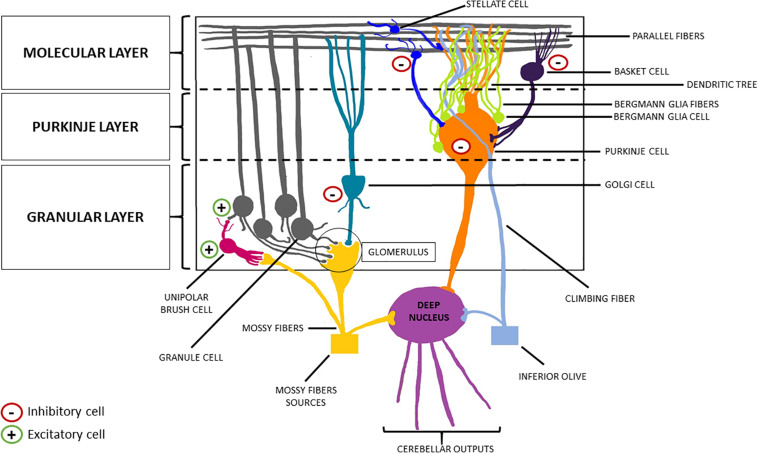
Schematic representation of the cerebellar cortex. From the innermost part to the outermost one, the cerebellar cortex can be divided into three layers: the granular layer, the Purkinje layer, and the molecular layer. The former welcomes the granule cells (excitatory neurons) and the Golgi cells (inhibitory interneurons). The descending dendrites of these two cellular types together with the ascending mossy fibers (originating from the brainstem nuclei, the spinal cord and the reticular formation) make synapsis in this area, forming the so-called “glomerulus”. Purkinje cells (inhibitory neurons) are located in the middle layer, from which they send their axon to the deep nuclei, crossing the granular layer, and their dendrites to the molecular layer, forming a “dendritic tree.” Finally, basket cells and stellate cells are the two inhibitory interneurons of the molecular layer, making synapsis with the parallel fibers (originating from the T-split of the ascending axons of the granule cells).

The cerebellar cortex is composed of three layers (from the deepest to the most superficial: the granular layer, the Purkinje layer, and the molecular layer), four inhibitory cell types [stellate cells, basket cells, Purkinje cells (PCs), and Golgi cells], two excitatory cell populations [granule cells and unipolar brush cells (UBCs)], and glial cells (among which Bergman glia; Figures 2, 3; Buffo and Rossi, 2013; Kandel et al., 2013; Roostaei et al., 2014).

The granular layer contains a large number of granular cells (excitatory). Each of them presents few descending dendrites, and a single ascending axon reaches the molecular layer, where it splits in a T-shaped way, generating the parallel fibers. This layer also contains interneurons, such as the UBCs and the Golgi cells, whose descending dendrites, together with the granule cells dendrites, and the mossy fibers (a major afferent cerebellar pathway originating from the brainstem nuclei, the spinal cord, and the reticular formation) form the synaptic structure known as “glomerulus” (Voogd and Glickstein, 1998; Mugnaini et al., 2011). Specifically, UBCs present one short dendrite whose brush engages in synaptic contact with a single mossy fiber terminal (the term brush indicates the fact that the tip of the UBC dendrite forms a paint brush-like tuft of dendrioles); while their axons branch locally within the granular layer, making contact with the granule cells (Mugnaini et al., 2011). Finally, the axons of the PCs (whose main body is located in the homonym middle layer, surrounded by multiple Bergman glia cells that, in turn, modulate their activity) cross this layer to reach the deep cerebellar nuclei, thus generating the cerebellar efferences (Buffo and Rossi, 2013). Conversely, the PCs send their dendrites to the molecular layer [wherein Bergmann glia (BG) fibers extend], forming a “dendritic tree,” making synapses both with the parallel fibers, and with the axons of the outer stellate cells (Voogd and Glickstein, 1998). These inhibitory interneurons are placed in the upper part of the molecular layer, as opposed to the internal stellate cells, which are below them and send an axon in the middle layer, making synapsis with the PCs (Sudarov et al., 2011; De Luca et al., 2016). Lastly, the molecular layer hosts the basket cells, another inhibitory interneuron type, and the climbing fibers. These latter represent the other major afferent pathway of the cerebellum, generating from the contralateral inferior olivary complex and making synapsis with the dendrites of PCs (Buffo and Rossi, 2013; Cattaneo, 2013; Roostaei et al., 2014).

Connecting the cerebellar cortex to the deep nuclei, there is the cerebellar white matter, namely the “arbor vitae,” so called for its tree-like branching pattern. It contains afferent and efferent fibers conveying sensorimotor information to/from the cerebellum (Cattaneo, 2013).

Finally, the deep nuclei constitute the cerebellar outputs, through which the fibers get to their targets after crossing the superior and inferior peduncles (the middle one is only crossed by afferents from the basilar pontine nuclei; Machold and Fishell, 2005; Wang et al., 2005). Nevertheless, it is worth recalling that the efferences going from the archicerebellum to the vestibular nuclei are the only ones that bypass the deep nuclei (Cattaneo, 2013; Roostaei et al., 2014).

Cerebellar circuitry appears to be a complex network of excitatory and inhibitory inputs and outputs with recurrent and interconnected loops involving the cerebellum and different brain regions. Notably, evidence deriving from anatomical, clinical, and neuroimaging data allowed us to point out the mutual connection between the cerebellum and basal ganglia, multiple cerebral cortex areas (particularly primary and associative ones), as well as thalamus and hypothalamus (Bostan et al., 2013; Benagiano et al., 2018).

This evidence showed that the cerebellum is not exclusively a motor structure, but it is part of somatotopically organized sensorimotor and cognitive networks, playing a significative role also in non-motor processes, such as cognition, visuospatial reasoning, associative learning, emotion, behavior, and, specifically in regard to basal-ganglia dense interconnections, reward-related learning (Schmahmann, 1998; O’Doherty et al., 2003; Stoodley et al., 2012).

Accordingly, patients with cerebrocerebellar damage are also showing specific cognitive disorders involving ideation (e.g., impairment of planning of daily activity), attention (e.g., impairment of shifting focus of attention), and sensory-perceptions (e.g., auditory or visuospatial neglect, sensory aphasia, dyslexia, and agnosia). Moreover, cerebrocerebellar damages have been detected in some patients affected by mood disorders or other psychiatric diseases (Benagiano et al., 2018).

## Embryology

To better perceive the cerebellar functions’ complexity, it is crucial to understand its morphogenesis and how its connections with other CNS structures are established. Cerebellar development is a process that starts early during the first trimester of pregnancy (30 days post- conception) and lasts until 2 years of postnatal age (van Essen et al., 2020). There are very few literature data regarding human cerebellar development before the 8th gestational week (Haldipur et al., 2018) and most of our knowledge about cellular and molecular maturation is derived from animal-based studies on fish, birds and rodents, especially mice. In view of the preservation of cerebellar ontogenesis and circuitry across evolution in these species, the analysis of these vertebrate models is crucial to understand the complexity of cerebellar development in the early stage of life (Haldipur et al., 2018, 2019). Even the correlation between volumes of the cerebellum and the total brain is consistent across species, though foliation is more variable in the hemispheres rather than the vermis (Leto et al., 2016).

The cerebellum originates from the dorsal portion of the hindbrain and its development can be summarized in four steps: organization of the cerebellar territory, establishment of cerebellar progenitors (GABAergic and glutamatergic ones), migration of the granule cells, and formation of the cerebellar nuclei and circuitry (ten Donkelaar et al., 2003).

Studies on chick, quail and mice embryos demonstrated that, at neural plate stage, the first segmental division is determined by the expression of the transcription factors Otx2 and Gbx2: the former defines the forebrain and midbrain territory, while the latter is expressed by the hindbrain, so their juxtaposition delineate the midbrain-hindbrain boundary (Marin and Puelles, 1994; Liu et al., 1999).

The isthmic organizer (IsO) is a patterning center that arises from this limit and expresses a protein of the fibroblasts growth factor family (FGF8), which is essential to the development and differentiation of cerebellum (Sato and Joyner, 2009). Other important products of the neural tube, such as WNT1, sonic hedgehog (SHH), bone morphogenetic protein, and transforming growth factor-ß, interact with the IsO to determine the anterior-posterior axis and the rhombomere segmentation (De Luca et al., 2016; van Essen et al., 2020).

In the upper part of the hindbrain, the rhombomere 1, the expression of the basic-helix-loop-helix proteins ATOH1 and PTF1a marks the rhombic lip (RL) and the ventricular zone (VZ), respectively, Hoshino et al. (2005), Machold and Fishell (2005), and Wang et al. (2005). In the lower portion of the RL, the most dorsal part develops into the roof plate, a transient pseudostratified epithelium constituted by a population of cells expressing the protein WNT1. This layer covers the fourth ventricle roof and originates the choroid plexus cells (Awatramani et al., 2003; Hunter and Dymecki, 2007). The choroid plexus is responsible for the production of the cerebrospinal fluid (CSF) and its proper function is essential for brain development: a reduction of CSF volume is implicated in cerebral growth impairment (Desmond and Jacobson, 1977; Andescavage et al., 2016), while its increase can determine hydrocephalus (McAllister, 2012). Moreover, an excess of CSF in the subarachnoid space has been proposed as an early marker of autism spectrum disorder (ASD; Shen et al., 2013). Interestingly, despite the cerebellum and the choroid plexus share a common genesis, Chizhikov et al. (2006) performed genetic fate mapping studies demonstrating that the roof plate does not originate from cerebellar cells.

Cerebellar nuclei cells are the first to be born, the glutamatergic ones originating from the RL and the GABAergic ones descending from the VZ and becoming interneurons (Haldipur et al., 2019). Development of the cerebellar nuclei starts with a “nuclear transitory zone” in a marginal position. The glutamatergic neurons follow a tangential pattern of migration and establish the GABAergic interneurons further maturation. The lateral nuclei develop early and project to thalamus and midbrain, while the medial group appears later and make connections to the hindbrain (Green and Wingate, 2014). Derivatives of the hindbrain that will form extra-cerebellar structures of the CNS also arise from the RL, like the pontine nuclei. A strict interconnection occurs between brainstem nuclei and cerebellum as the brainstem delivers proprioceptive/vestibular/auditory sensations and cortical information to the cerebellum (Machold and Fishell, 2005; Wang et al., 2005). In the developing murine brain, the protein semaphorin 3A acts as a guidance for the pontocerebellar axons that will reach the cerebellar granule layer and form the mossy fibers (Solowska et al., 2002).

Rhombic lip produces all the remaining cerebellar glutamatergic interneurons and neurons. The excitatory interneurons are the UBCs, especially represented in the flocculonodular lobe (Munoz, 1990; Víg et al., 2005). The granule layer cells are the glutamatergic neurons and spring up from granule cell progenitors (G). This population migrates tangentially to form the external granule layer (EGL) and, following FGF8 and SHH signaling, goes through clonal expansion during the late pregnancy period, determining the formation of a six-eight cells layer (Sato and Joyner, 2009). This process produces an amount of granule neurons so large that it overcomes the cerebral cortex ones (Roostaei et al., 2014; Leto et al., 2016). Later, GCPs differentiate and move inward into the cerebellar anlage to form the internal granule layer (IGL). During the postnatal age, the RL continues to produce granule cells and the EGL progressively disappears during the second year (Rakic and Sidman, 1970; Haldipur et al., 2018; van Essen et al., 2020).

The VZ originates the GABAergic neurons (PCs) and interneurons of the cerebellum. Two groups of PCs leave the VZ to form the so-called “Purkinje cell plate” (Goffinet, 1983): early PCs, born in the posterior VZ, migrate tangentially, then change orientation toward the EGL under the influence of the protein reelin secreted by the GCPs; on the contrary, late PCs, born in the anterior VZ, move following a radial pattern, guided by Bergmann glial fibers signaling (Yamada and Watanabe, 2002). Subsequently, the PCs plate reorganizes itself to form a monolayer of PCs, beneath which the IGL will locate (Ben-Arie et al., 1997; van Essen et al., 2020). The granule cells produce trophic factors necessary to develop the PCs dendritic trees. At 20th gestational week, the human cerebellum presents a transient cellular region called “lamina dissecans,” between the PCs layer and the IGL. Its function in the cerebellar development is yet unknown and it disappears by the 32nd gestational week (Rakic and Sidman, 1970; Haldipur et al., 2018). All inhibitory interneurons come from a common progenitor expressing a protein called PAX2 and, during the third trimester of pregnancy, differentiate in Golgi cells, that will establish in the granule layer, and stellate and basket cells, that will take place in the molecular layer (Sudarov et al., 2011).

Cerebellar astrocytes, BG and a small number of oligodendrocytes expressing Olig2 domain originate from the VZ (Seto et al., 2014), while the majority of oligodendrocytes derive from extracerebellar regions of the CNS (Lee et al., 2005). Glial cells are involved in numerous processes of the cerebellar development: cellular migration (especially the PCs), synapse organization, production of neurotrophic factors, and formation of the blood-brain barrier (Araujo et al., 2019).

In mice, the exponential proliferation of the GCPs occurs after birth, while, in humans, it starts at 24th gestational week and continues during postnatal age, achieving a peak at the 32nd gestational week (Dobbing and Sands, 1973; Volpe, 2009). This process provokes an increase in the cerebellar mass that exceeds the volume of the posterior fossa, determining a series of folding along the anterior/posterior axis and allowing the expansion of the cerebellar surface (Sudarov and Joyner, 2007). This foliation starts with the organization of the “anchoring centers” and the appearance of scissures that form the folia and separate the lobes. In this process equally participate EGL, PCs, and BG (Sudarov and Joyner, 2007).

## Cerebellar Neuroimaging

Neuroimaging studies on the cerebellum have provided important insights into its role both in adulthood and developing age. Continuous spreading and implementation of non-invasive neuroimaging tools have led us to: (1) monitoring the structural and functional state of the cerebellum, (2) detecting eventual abnormalities, (3) making diagnosis, (4) assessing prognosis, and (5) suggesting and monitoring therapies (Manto and Habas, 2016).

Magnetic resonance imaging (MRI), performed through both conventional (such as T1, T2, proton density, FLAIR, and DWI) and unconventional methods (such as functional MRI techniques), represents the most supportive diagnostic tool (Currie et al., 2013).

The recent advances in prenatal diagnosis allowed to monitor the development of the cerebellum during fetal life and early detect several malformations (Plaisier et al., 2015; D’Antonio et al., 2016). Furthermore, thanks to the recent developed complementary tools, structural or anatomical abnormalities may be detected and metabolic and functional ones (D’Antonio et al., 2016).

### Functional MRI Techniques

Among the functional MRI techniques available, it is worth mentioning blood oxygenation level-dependent functional magnetic resonance imaging (BOLD-fMRI), resting-state functional connectivity, MR spectroscopy and diffusion tensor imaging (DTI; Manto and Habas, 2016).

Blood oxygenation level-dependent functional magnetic resonance imaging is a technique that specifically exploits the differences in blood flow among the brain regions to compose functional images based on the increased activity of a specific site during a task (Glover, 2011). Structural and/or functional damages of certain areas of the CNS, therefore, of the cerebellum, may generate abnormal signals, contributing to giving important information about cerebellar diseases (Manto and Habas, 2016). Jayakumar et al. (2008) performed a BOLD-fMRI on SCA1 patients and healthy patients during the execution of the hands’ supination-pronation movements, detecting an activation of cerebellum network during this movement and a malfunctioning of cortico-cerebellar loops in the former. Another study carried out by Duarte et al. (2016) used BOLD-fMRI to compare SCA3 patients versus a control group while performing a motor task (finger tapping). The authors demonstrated the cerebellum’s abnormal response signals and other cerebral regions, such as the somatosensory and prefrontal cortices and subcortical areas (putamen, globus pallidus, and thalamus). According to their work, this technique may allow not only to discriminate between SCA3 patients and controls executing the motor task at 5 Hz, but also to monitor the disease progression through performance-level-dependent differences.

Resting-state fMRI methods are based on the assumption that different brain regions, sharing a temporal correlated activity in terms of spontaneous BOLD signal, form functional networks that can be analyzed in healthy subjects and pathological conditions. They appear to be promising techniques in detecting the brain’s functional alterations, such as in ataxias, and, interestingly, in psychiatric disorders too (Woodward and Cascio, 2015). This is even more intriguing in the light of the fact that this particular tool, compared to other fMRI techniques, does not require the patient’s active participation (Woodward and Cascio, 2015). Khan et al. (2015) exploited this peculiar technique to assess functional cortico-cerebellar connectivity in autistic children and adolescents, detecting an atypical predominant cortico-cerebellar overconnectivity, majorly in sensori-motor networks, and a concurrent underconnectivity in supramodal ones. A later study carried out by Ramos et al. (2019) on a vast cohort of autistic patients provided further data, detecting a reduced intrinsic connectivity between the cerebellum and various cortical regions involved in ASD, i.e., right fusiform gyrus, right superior temporal gyrus, and left middle temporal gyrus. MinlanYuan, Meng et al. (2017) used resting-state fMRI to demonstrate the relationship between social anxiety disorder and specific cerebellar neural circuits, revealing lower levels of connectivity in certain cerebellar regions of patients than controls. Furthermore, this study allowed to correlate higher levels of anxiety to lower connectivity and to predict clinical improvement after cognitive-behavioral therapy in those patients with stronger circuits levels at baseline. Though the relatively small cohort of patients, this study is consistent with the aforementioned concept that functional neuroimaging is slowly becoming more crucial in clinical practice (MinlanYuan, Meng et al., 2017).

H-magnetic resonance spectroscopy is used to measure quantitatively and qualitatively numerous neurometabolites, among which *N*-acetyl-aspartate, Choline, Creatine, Myo-inositol, Lactate, Glutamate, Glutamine, and Gamma-aminobutyric acid (GABA), possibly contributing to various brain diseases and widely implicated in neuronal and axonal health, membrane turnover, energetic metabolism, and neurotransmission (Zhu and Barker, 2011).

Concerning cerebellar disorders, this technique seems particularly useful when it comes to tumors, infections, strokes, metabolic disorders, or white-matter diseases, as well as in the assessment of the metabolism of other organs, such as heart and muscle, often compromised in cerebellar ataxias (Lodi et al., 2001; Nachbauer et al., 2013; Manto and Huisman, 2018).

Diffusion tensor imaging is a non-invasive MRI technique based on the analysis of diffusion of water in different tissues, allowing a three-dimensional (3D) graphic reconstruction of the areas investigated, permitting, and a 3D analysis of white matter. In this latter case, the method is also referred to as tractography (Habas and Manto, 2018). This technique is particularly interesting when it comes to cerebellar disorders, since it provides important information on its anatomy and its connections (afferents and efferences), even during the phase of the development. In this regard, Bruckert et al. (2019) retrospectively analyzed the MRI tractography scans of a vast cohort of patients (aged 1 day to 17 years) showing age-related changes of the cerebellar white matter throughout the development, and reporting, an increase of mean tract fractional anisotropy and a concomitant decrease of mean tract mean diffusivity in all peduncles, with particularly rapid changes both in diffusion measures during the first 24 months of life, followed by more gradual change later in life. Despite the limitations of the study, e.g., the small sample of subjects and the interindividual differences among them, this work offers new interesting data that may contribute to identifying sensitive biomarkers of cerebellar abnormalities and better characterize cerebellar circuitry in clinical populations (Bruckert et al., 2019).

Moreover, as recently reviewed by Crippa et al. (2016), different studies showed a decrease of fractional anisotropy, measure of white matter integrity, in the white matter of individuals with ASD, hence, suggesting a weaker structural connectivity of their cerebro-cerebellar circuits, and strengthening the currently accepted notion of ASD as a connectivity disorder.

In conclusion, tractography can be used to study cerebellar development since fetal life, particularly in preterm brain injuries and neurodevelopmental disorders in which the cerebellum is notably often involved. Habas and Manto (2018) recently reviewed previous reports showing the different tractography applications in cerebellar diseases, such as cerebellar malformations, autosomal-recessive ataxias, neoplastic or degenerative disorders, and traumatic injuries, throughout the different phases of life.

### Other Neuroimaging Techniques

A separate mention should be made for other neuroimaging techniques, such as nuclear medicine and ultrasonography.

Single-photon emission computed tomography and positron emission tomography (PET) must be cited, though their application to the pediatric field remains limited due to the use of dangerous radiation (Manto and Huisman, 2018). Nevertheless, Szyszko et al. (2015) have recently described a potential role of F-fluorodeoxyglucose PET/computed tomography in the assessment of certain pediatric dystonia, such as neurodegeneration with brain iron accumulation, in which an overactivity of the putamen and an underactivity of the cerebellum were detected.

Cranial Ultrasound Scan (cUS) is a common diagnostic tool, safe and feasible, allowing bed-sided serial investigations in newborns (Ecury-Goossen et al., 2015). cUS appears particularly useful for the evaluation of the neonatal brain, especially for the identification of those at high risk of neurodevelopmental impairment, such as the preterm ones (Plaisier et al., 2015; Steggerda et al., 2015). cUS has two main limits: it is highly related to the rater’s skills, and, it is applicable only until the acoustic windows are available (approximately 6 months of age; Steggerda and van Wezel-Meijler, 2016). The best acoustic window to study the posterior fossa is usually represented by the mastoid fontanel (MF; Buckley et al., 1997; Luna and Goldstein, 2000; Steggerda et al., 2015). However, a recent study carried out by Muehlbacher et al. (2020) encouraged the approach through the Foramen occipital magnum (FOM) in very low birth weight infants. The FOM window allows to examine both hemispheres at the same time and easily detect even small cerebellar damages. Therefore, this approach must be considered, especially in very preterm infants (Muehlbacher et al., 2020). Particularly in this specific population, different studies proved that cUS, though unable to detect cerebellar microhemorrhages, plays an important role in revealing massive cerebellar ones, severe conditions potentially leading to cerebellar disruption and eventual atrophy, with subsequent long-term pervasive neurodevelopmental impairments, involving cognitive, learning, and behavioral areas (Limperopoulos et al., 2005, 2007; Parodi et al., 2015). Finally, it may be concluded that recent developments in the ultrasonography field, such as contrast-enhanced ultrasonography, microvessel imaging, or elastography, are significantly enriching the neonatal care field (Manto and Huisman, 2018).

## Overview of the Cerebellar Functions: From Motor Tuning to Cognition

Originally, the cerebellum was thought to be merely implicated in motor functions, as it is involved in maintaining balance and in executive control of movements (synergy), almost functioning as a “real-time movement tuner.” Therefore, a lesion of the cerebellum usually presents with gait and balance disorders (namely ataxia and astasia, respectively) and abnormal coordination of opposite muscle groups (asynergia), leading to an impaired coordination of the limbs (dysmetria), difficulties in articulation of speech (dysarthria), and excessive movements of the eyes with inadequate focus stability (nystagmus; Mutani et al., 2012; Schmahmann, 2019).

Since the attention to cerebellar malfunction shifted from the motor disorders to the cognitive and behavioral impairments, we discovered the numerous non-motor roles of this complex organ, especially memory, executive functions, and language (Sathyanesan et al., 2019). This is consistent with the data suggesting that most of the cerebellum is connected with associative areas of the cerebral cortex (Bostan et al., 2013).

Several cerebellar functions proposed during the last few decades included: motor and sequence learning, biological timer, prediction of results and movement assessment, automatization, long-term depression supporting memory, body dynamics storage and sensory inputs integration (Schmahmann et al., 2019). Schmahmann proposed a theory based on the repetitiveness of the cerebellar microanatomical structure, the universal cerebellar transform (UCT). The UCT acts as an unconscious regulator of movement and behavior, combining external and internal stimuli adjusting the response to the circumstances. According with this assumption, deficits of non-motor functions are described as “Dysmetria of thought” (Schmahmann, 1998). This hypothesis is also interesting in the perspective of the cerebellum, seen as a fundamental organ engaged in the development and specialization of cognitive regions of the cerebral cortex (Molinari et al., 2018).

As previously discussed, the cerebellum has been divided into three functional areas: the anterior lobe, formed by lobules I–V, is mostly implicated in sensorimotor connections and in determining posture, tone e movements; the posterior lobe, represented by lobules VI–IX, is involved in cognitive, social and behavioral tasks; the flocculonodular lobe, constituted by lobule X, is responsible for equilibrium, eye movements and adjusting reflexes (Stoodley and Schmahmann, 2018). These findings have been confirmed by numerous anatomic, clinical and functional neuroimaging studies, demonstrating no overlap between motor and cognitive/affective areas except for tasks that include both motor and non-motor characteristics, such as language and social processing (Sathyanesan et al., 2019; Schmahmann et al., 2019).

Among the cognitive functions fulfilled by the cerebellum, there are visuomotor sequence learning (the ability to recognize events and organize them as a sequence) and other executive functions, such as visual and auditory sequential memory, strategy planning, visuo-spatial ability, and attention (Molinari et al., 1997; Riva, 1998). These functions are implicated in writing and reading; therefore, a lesion of posterior regions can determine learning disabilities and other neurodevelopmental disorders (Vicari et al., 2005). Also, the behavior is affected by cerebellar lesions, indicating an involvement in its regulation, and ataxic patients can present with an excess or reduction of response to internal and external stimuli (Molinari et al., 2018).

Language is one of the most important functions in which the cerebellum is implicated. Considering the initial belief of a mere involvement in the motor control of speech, to date, a more complex role of the cerebellum, including non-motor aspects of language, has emerged (Mariën et al., 2014). An involvement of the right lateral region of the cerebellum, contralateral to the Broca’s area, has been widely demonstrated in language impairments through anatomical and functional neuroimaging studies (Mariën and Borgatti, 2018). In detail, a lesion in the right cerebellum may, in its turn, determine a functional depression of supratentorial language areas due to reduced inputs crossing the cerebello-cortical pathways (cerebello-cerebral diaschisis; Mariën et al., 2009). Conversely, a cerebro-cerebellar diaschisis, namely a supratentorial lesion causing a loss of function in cerebellar regions, has been associated with language deficits (Abe et al., 1997). Furthermore, today there is new evidence of a more intricate connection between both cerebellar hemispheres and dominant language cortex, supporting a possible left cerebellar hemisphere contribution to linguistic processes *via* ipsilateral cerebellar-basal ganglia-cortical pathways, hence claiming a role of ipsilateral cerebello-cerebral diaschisis in language impairments (Murdoch and Whelan, 2007).

The cerebellum’s association with language function is determined by the specific cortico-cerebellar connectivity to the right cerebellum from the left cortical hemisphere, and supported by structural and functional connectivity analyses that revealed projections from higher-order association areas, including the prefrontal, posterior parietal, and superior temporal cortices, known to be involved in language function, to posterior cerebellar lobules VI, Crus I, Crus II, and VIIb (Figure 1). Further studies are needed to well-assess these complex projections and establish a functional topographic map (especially to lead specific targets for rehabilitation; Vias and Dick, 2017).

There are many signs and symptoms related to speech in children, including deficits in fluency and verbal initiative, anomia, grammar, and pragmatical difficulties, or even total absence of language (Salman and Tsai, 2016). An interesting entity is the cerebellar mutism syndrome (CMS), consisting of a temporary total absence of speech, except for vocal phenomena such as whining and crying, due to various cerebellar damages, such as after surgical rescission of cerebellar tumors (Catsman-Berrevoets, 2017).

Still, in the posterior lobe, social functions are localized in the vermis zone, especially the ability to process emotion, i.e., pain, thanks to its connections to the limbic lobe. On the contrary, more complex social skills, as empathy, theory of mind, abstraction and mentalization are thought to be localized in the paravermial lobules (Leggio and Olivito, 2018).

Furthermore, recent functional neuroimaging findings comparing subjects with typical and atypical development (autism spectrum disorder) revealed an abnormal connectivity between the cerebellum and specific cortical areas in charge of functions usually compromised in autism, therefore strictly linked to autistic traits and symptoms, such as abnormal social processing (fusiform gyrus and lateral temporal cortex), motor impairments and stereotyped behaviors (frontocerebellar pathways; Just et al., 2012; Floris et al., 2016; Ramos et al., 2019). In this regard, Rogers et al. (2013) emphasized the association between abnormalities of cerebellar functions in autism and deficits in cognitive, motor behavior, and social reward. Moreover, though much more has yet to be unraveled, Right Crus I (RCrusI; Figure 1) dysfunctions have been reported both in mice models and humans with autism. In fact, this region is considered functionally related to circuits implicated in autism; hence a dysfunction of RCrusI may determine autistic symptoms, particularly in terms of social impairments and repetitive behaviors. Nevertheless, further data are needed to clearly assess its exact contribution to ASD and its eventual causal relationship with potential coexistent motor deficits (Stoodley et al., 2017). In conclusion, it is worth mentioning that cerebellar neuroanatomical alterations are the most replicated findings in postmortem brain samples of patients with autism (Wegiel et al., 2014).

## Cerebellar Neurological Findings

Several clinical signs can be evaluated when trying to assess cerebellar functioning. In this section we will therefore analyze detailed neurological findings according to an age-related assessment, thus retracing cerebellar physiological functions and development.

Abnormal eye movements are very common in cerebellar lesions. Nystagmus and inappropriate saccades are consistent findings of the so-called ocular instability. Five types of nystagmus have been described in the cerebellar oculomotor syndrome: (1) the downbeat nystagmus (upward slow phase and downward fast-phase of nystagmus); (2) gaze-evoked nystagmus (when the fast-phase corresponds to the direction of gaze); (3) periodic alternating nystagmus (a spontaneous horizontal nystagmus with periodic alternation of direction); (4) positioning nystagmus (which changes in relation to different head positions); and (5) spontaneous and head-shaking nystagmus (which follows head oscillations; Bodranghien et al., 2016). Inappropriate saccades usually consist of hypometric or hypermetric saccadic eye movements, meaning that the patient may under-, or overshoot the fixation target (Ataullah and Naqvi, 2020). Nevertheless, other deficits in saccades may be detected in cerebellar patients, as well as further ocular impairments, such as ocular misalignment and impaired vestibulo-ocular reflex (Bodranghien et al., 2016). Overall, even if not always anatomically specific, the above-mentioned oculomotor deficits can represent the only evident cerebellar abnormality in the early age of life (Weiss et al., 2009; Lee et al., 2016).

As cerebellar is involved in tone regulation (tonic function), hypotonia is usually encountered in hemispheric cerebellar lesions. Cerebellar patients often have pendular reflexes resulting in oscillations of the limb at the percussion and abnormal “dampening.” This is due to the impaired calibration of muscular contraction (Mutani et al., 2012; Ataullah and Naqvi, 2020). Conversely, patients with chronic cerebellar syndromes usually show hypertonia, probably due to other brain systems’ involvement. Cerebellar ataxic gait is typically characterized by a widened base, unsteadiness and clumsiness, with poor coordination of the legs and feet (usually excessively raised at each step), and lateral veering, and it is often referred to as “drunken” gait (Mochizuki and Ugawa, 2010; Ataullah and Naqvi, 2020). To assess the steadiness of gait, it may be interesting to test tandem gait too, asking the patient to walk ideally in a straight line, in such a way that the heel of the front foot touches the toes of the back one at each step. Usually, cerebellar ataxic patients are not able to do so. Furthermore, in a recent metanalysis, Buckley et al. (2018) have summarized the main differences between gait of cerebellar ataxic patients and healthy controls. It appears that the former group, in comparison to the latter one, presents a reduction of walking speed, cadence, step length, stride length and swing phase and an increase of base width, stride time, step time, stance phase and double limb support phase, as well as a higher variability of step length, stride length and stride time (Buckley et al., 2018). The above-mentioned peculiar characteristics are consistent with the typical walking style of ataxic patients previously mentioned (Mochizuki and Ugawa, 2010; Ataullah and Naqvi, 2020).

In regard to balance, besides the tandem gait, the Romberg test should be performed too. This test is considered positive when the patient, with the eyes closed, loses his balance, oscillating or falling. It is noteworthy mentioning that cerebellar ataxic patients usually present with a negative Romberg test. However, this peculiar maneuver is useful for a differential diagnosis, allowing to detect sensory ataxia (positive Romberg test), hence indicating the need to investigate the presence of myelopathies (Forbes and Cronovich, 2020). Cerebellar ataxia, due to vermian lesions, is usually characterized by continuous oscillation of the body, retropulsion and attempts to correct muscular contractions, both with eyes open and closed (Mutani et al., 2012).

Vertigo and dizziness (presenting with an acute onset, or as recurrent attacks, or as chronic/permanent signs) are usually associated with imbalance and may be due to lesions of the cerebellar ocular motor systems or the pathway related to the vestibulocerebellum (Bodranghien et al., 2016).

To assess the presence of asynergia, the rebound phenomenon must be tested. This is useful to assess the inability of the malfunctioning cerebellum to regulate the action of opposing muscle groups. The examiner applies a certain pressure against the resistance exerted by the arm of the patient (outstretched or flexed with a closed fist), releasing it suddenly. Normally, a fleeting oscillation and a rapid return to the original position is expected. However, in cerebellar dysfunctions, a strong, inappropriate, rapid and opposite muscular force generates when the tested arm is released (Ataullah and Naqvi, 2020).

Cerebellar asynergia can be also evaluated by the finger-nose test and the heel-shin one. These tests may reveal other important cerebellar signs, such as hypermetria (when too much strength is used to reach the targets, namely nose, or shin) or dysmetria (when the finger/heel overshoots the target). Furthermore, cerebellar patients usually tend to decompose every single movement in its subparts and, when asked to alternate opposite movements rapidly, they are unable to do it (dysdiadochokinesia; Mutani et al., 2012; Ataullah and Naqvi, 2020).

Another important neurological sign of motor dysfunction due to cerebellar lesions is “intention tremor” or “action tremor” which starts and progressively increases in amplitude during the terminal phase of voluntary movement, disappearing during rest (Mutani et al., 2012; Ataullah and Naqvi, 2020). Even though not always tested, reaching and grasping movements are often compromised in cerebellar patients.

This may be secondary to other cerebellar signs and symptoms (such as tremor, decomposition of movements, dysmetria, and abnormal eye-movements), or directly related to a specific cerebellar damage (such as atrophy). Finally, language, cognitive and affective functions should also be assessed since they may be compromised in cerebellar damages, thus determining the previously mentioned CCAS (Bodranghien et al., 2016).

Cerebellar patients may present executive function deficits (with inability to plan and organize, poor problem-solving capacity, and concrete thinking), impairments of short-term memory, and visual-spatial deficits (Bodranghien et al., 2016).

Expressive language is often abnormal. “Slurred staccato speech,” also known as “scanning speech,” is a common finding in cerebellar disorders (Ataullah and Naqvi, 2020). This type of language is characterized by reduced verbal fluency, with words pronounced as broken in syllables and with long pauses between them. Moreover, reluctance to engage in conversation, difficulty in finding words, abnormal syntax and impaired metalinguistic ability may occur (Bodranghien et al., 2016). Recently, mutism of cerebellar origin (namely the aforementioned CMS) has been reported too (Catsman-Berrevoets, 2017).

Finally, with respect to the affective aspects, cerebellar damage may determine a wide variety of impairments, with regard to mood and emotional regulation, personality style, behavioral modulation, and social skills.

Moreover, nowadays, cerebellar lesions may correlate with neurodevelopmental and psychiatric disorders, such as autism, psychosis, mood disorders, panic disorder, dyslexia, attention deficit disorder, and many others (Bodranghien et al., 2016; MinlanYuan, Meng et al., 2017).

Overall, lesions of the anterior lobe appear to cause sensorimotor impairments, whereas lesions of the posterior lobe are associated with CCAS (Schmahmann et al., 2019).

## Age-Related Cerebellar Examination

The cerebellum in childhood may subserve several age-dependent functions that can be affected in a variety of CNS pathologies. An age-related assessment in such patients may address early diagnosis and proper intervention since the first months of life (Haldipur and Millen, 2019).

In newborns and infants, the neurological examination may be conducted using standardized methods, such as the Hammersmith Neurological examination, both in its Neonatal and Infant version (HNNE and HINE, respectively; Di Rosa et al., 2016). To the best of our knowledge, although correlations between HINE and HNNE specific items and cerebellar development have not been reported yet, hypotonia, developmental delay, abnormal reflexes, and abnormal eye movements are frequently observed in newborns and infants with cerebellar malformations (Romani et al., 2013). Feeding difficulties and failure to thrive are commonly related to hypotonia or uncoordinated oro-motor function in these children and sometimes require nasogastric feeding tubes or gastrostomy placement to prevent aspiration and provide adequate caloric intake. Abnormal ocular movements (oculomotor apraxia, nystagmus, and strabismus) can be early onset features associated with cerebellar malformations and potentially represent the most suggestive “cerebellar” findings in newborns or infants. Fine-motor skills are commonly affected in children with cerebellar dysfunctions, with subsequent age-related impairment in reaching, grasping, visuomotor manual skills, drafting and writing across infancy and early childhood. Indeed, clinical evaluation of balance and gait, cannot be easily performed before 18 months of age, when independent standing and walking have already appeared in the majority of children (Scharf et al., 2016).

Tam et al. (2019) recently reported the constellation of neurological findings in a cohort of preterm infants with cerebellar hypoplasia. Hypotonia was the most common finding at 18 months’ corrected age, associated with hyperreflexia and postural instability on standing, likely related to smaller cerebellar volume (Tam et al., 2019). Oculomotor apraxia is a highly suggestive symptom of cerebellar malformation also in toddlers and older children. Saccadic initiation failure is the most common eye movement abnormality, being saccades hypometric or absent (Tusa and Hove, 1999; Salman and Chodirker, 2015). Furthermore, more complex abilities, such as tandem gait or finger-nose and heel-shin tests, cannot be examined before 4 years of age or according to the cooperation skills of the child. As a rule, in children aged between 4 and 8 years, specific cerebellar tests (particularly those exploring fine-coordination skills) may be physiologically affected by the degree of cerebellar maturation, as well as the cooperation of the patient. After 8 years of age, a complete neurological examination may be performed as well as in the adult age. The most common motor symptoms of cerebellar dysfunction in school-aged children include dyssynergia, dysmetria, dysdiadochokinesia, and ataxia. Expressive language impairment with scanning speech due to oral-motor dyspraxia can be easily disclosed in preschool and school-aged children. The neurological signs of cerebellar dysfunctions across developing age are summarized in Table 1.

**TABLE 1:** Age related signs and symptoms in cerebellar malformations.

Age-onset	Hypotonia	Ocular symptoms	Coordination disorders	Oro-motor apraxia	Balance disturbances	Ataxia	Other cerebellar signs
Neonatal	+++	++	+++	+ –	/	/	/
Infantile	++	+++	+++	+++	/	+−	/
Prescholar	+−	+++	++	++	+−	+++	++
School-age	+−	+++	+−	+−	+++	+++	+++

*Signs or symptoms severity: / = not valuable; +–: not always valuable; + = mild severity; ++ = moderate severity; and +++: severe.*

## Discussion

The cerebellum remains a fascinating and still relatively unexplored part of the CNS involved in several neurocognitive functions. From the initial mere involvement in motor coordination up to a central role in cognition, social behavior, and communication, we were led to revalue the developmental role of the cerebellum. Age-dependent neurological findings need to be taken into account in clinical practice to assess cerebellar functioning even in early childhood properly. Even though the main evidence of cerebellar involvement comes from the observation of the pathological counterpart, fewer insights have been provided by longitudinal studies in typically developing children. Further studies in wide populations may provide details on clinical developmental trajectories of cerebellar functions and address early detection of pathological conditions.

## Author Contributions

AN and GD conceived, planned, and supervised the study. GA and GS wrote the first draft of the manuscript. GA and AI designed the figures. LV and GQ helped supervise the project. All authors contributed to manuscript revision, read, and approved the submitted version.

## Conflict of Interest

The authors declare that the research was conducted in the absence of any commercial or financial relationships that could be construed as a potential conflict of interest.
